# Supplementary material for: Facilitating the access to HIV testing at lower costs: “To the laboratory without prescription” (ALSO), a pilot intervention to expand HIV testing through medical laboratories in France
Source: PLoS One. 2024 Oct 24;19(10):e0309754. doi: 10.1371/journal.pone.0309754 (PMC11500895; doi:10.1371/journal.pone.0309754)
Supplement: S1 Table — (DOCX) [file pone.0309754.s001.docx]

**S1 Table. Characteristics of users according to the type of testing, ALSO or STI clinics, On-site survey, November 2019 and November 2020.**

|  |  | 2019 | | | 2020 | | |
| --- | --- | --- | --- | --- | --- | --- | --- |
|  |  | ALSO tests | STI clinics |  | ALSO tests | STI clinics |  |
|  |  | N=295 | N=711 |  | N=573 | N=388 |  |
|  |  | % | % | p-value | % | % | p-value |
| County | Paris | 67 | 89 | <0 .001 | 64 | 93 | <0.001 |
|  | Alpes-Maritimes | 33 | 11 |  | 36 | 7 |  |
| Group | Women | 42 | 40 | <0.004 | 43 | 33 | <0.001 |
|  | Heterosexual men | 42 | 35 |  | 41 | 34 |  |
|  | Men who have sex with men | 15 | 24 |  | 16 | 31 |  |
|  | ND | 1 | 1 |  | 1 | 2 |  |
| Age | Age (years), median (IQR) | 32 (25-43) | 26 (22-32) | <0.001 | 35 (26-44) | 26 (22-31) | <0.001 |
| Education | <High school | 13 | 8 | 0.008 | 13 | 7 | 0.01 |
|  | High school | 15 | 21 |  | 17 | 19 |  |
|  | University (2 years) | 16 | 13 |  | 14 | 14 |  |
|  | University (≥3 years) | 54 | 56 |  | 55 | 59 |  |
|  | ND | 2 | 2 |  | 4 | 1 |  |
| Activity | Employed | 70 | 48 | <0.001 | 75 | 56 | <0.001 |
|  | Unemployed | 9 | 12 |  | 7 | 11 |  |
|  | Student | 14 | 32 |  | 11 | 29 |  |
|  | No activity/retired | 5 | 4 |  | 5 | 2 |  |
|  | ND | 1 | 4 |  | 2 | 3 |  |
| Place of living versus place of testing | Same department | 71 | 53 | <0.001 | 76 | 61 | <0.001 |
|  | Same region | 19 | 33 |  | 18 | 30 |  |
|  | Other region or abroad | 9 | 11 |  | 5 | 8 |  |
|  | ND | 1 | 3 |  | 1 | 1 |  |
| Place of birth | France | 79 | 72 | 0.031 | 80 | 75 | 0.14 |
|  | Overseas French regions | 3 | 2 |  | 2 | 3 |  |
|  | Abroad | 18 | 26 |  | 15 | 20 |  |
|  | ND | 0 | 1 |  | 2 | 2 |  |
| History of testing | Previous test | 81 | 74 | 0.027 | 84 | 79 | 0.20 |
|  | First test | 17 | 25 |  | 15 | 20 |  |
|  | ND | 2 | 1 |  | 1 | 1 |  |
| N. of sexual partners over the last 12- | 0 or 1 | 29 | 16 | <0.001 | 23 | 15 | <0.001 |
| month period | ≥2 | 60 | 79 |  | 69 | 80 |  |
|  | ND | 11 | 5 |  | 8 | 5 |  |
| HIV exposures in the previous 5 years | None | 81 | 67 | <0.001 | 81 | 67 | <0.001 |
|  | 1 | 16 | 25 |  | 16 | 25 |  |
|  | ≥2 | 3 | 8 |  | 3 | 8 |  |
| Ever feared to be HIV infected | No | 53 | 42 | 0.004 | 46 | 45 | 0.6 |
|  | Yes | 39 | 47 |  | 45 | 47 |  |
|  | ND | 8 | 11 |  | 17 | 15 |  |
| Health insurance | Regular insurance | 88 | 79 | <0.001 | 83 | 78 | 0.008 |
|  | AME or none | 6 | 15 |  | 6 | 12 |  |
|  | ND | 5 | 6 |  | 11 | 10 |  |
| Visit to GP/12 months | None | 6 | 10 | 0.3 | 6 | 9 | 0.4 |
|  | 1 visit | 25 | 24 |  | 21 | 21 |  |
|  | >1 visit | 63 | 60 |  | 62 | 60 |  |
|  | ND | 6 | 6 |  | 11 | 10 |  |

ND: missing data or do not wish to answer. AME for “Aide Médicale d’Etat”: provisory health insurance offered to undocumented migrants
