# Supplementary material for: Facilitating the access to HIV testing at lower costs: “To the laboratory without prescription” (ALSO), a pilot intervention to expand HIV testing through medical laboratories in France
Source: PLoS One. 2024 Oct 24;19(10):e0309754. doi: 10.1371/journal.pone.0309754 (PMC11500895; doi:10.1371/journal.pone.0309754)
Supplement: S3 Table — (DOCX) [file pone.0309754.s003.docx]

**S3 Table. Mean costs of HIV testing, by step and in total, according to test results, estimated by microcosting for an ALSO test carried out in laboratory (lab)**

| **ALSO – NEGATIVE HIV test** | **(€)** |  | **ALSO – POSITIVE HIV test** | **(€)** |
| --- | --- | --- | --- | --- |
| **HIV testing in lab** | **12.57** |  | **HIV testing in lab** | **72.27** |
| Admission | 0.70 |  | Admission | 0.70 |
| Blood sampling | 4.43 |  | Blood sampling | 4.43 |
| Transport of sample from sampling site to analysing site | 0.59 |  | Transport of sample from sampling site to analysing site | 0.59 |
| Combined ELISA/AgP24 analysis | 4.67 |  | Combined ELISA/AgP24 analysis | 4.67 |
| Western blot analysis ^1^ | 0.11 |  | Western blot analysis | 45.97 |
| Result validation | 1.88 |  | Result validation | 1.88 |
| Result delivery (mail, email) | 0.20 |  | Result delivery by the biologist or information of GP about the positive result ^2^ | 14.03 |
| **Mean cost for one negative HIV ALSO test** | **12.57** |  | **Confirmatory analysis on a second sample** | **12.27** |
|  |  |  | Admission | 0.70 |
|  |  |  | Blood sampling | 4.43 |
| ^1^ The specificity of combined ELISA/ AgP24 detection being 99.8%, a Western blot was carried out for 0.2% of negative tests.  ^2^ The biologist delivered the result in 2/3 of positive tests and refered to the GP in 1/3 of the positive tests.  ^3^ In 1/3 of the positive tests, HIV diagnosis delivery by GP is considered as complex consultation, the cost of a GP visit of €25 is increased by €30.  ^4^ First HIV care consultation is considered as complex consultation, the cost of a specialist physician visit of €30 is increased by €30. |  |  | Transport of sample from sampling site to analysing site | 0.59 |
|  |  |  | Combined ELISA/AgP24 analysis | 4.67 |
|  |  |  | Result validation | 1.88 |
|  |  |  | **Medical consultation (GP) for positive result delivery ^3^** | **18.33** |
|  |  |  | **Consultation to a specialised HIV unit^4^** | **60.00** |
|  |  |  | **Mean cost for one positive HIV ALSO test** | **162.86** |
