# Supplementary material for: Facilitating the access to HIV testing at lower costs: “To the laboratory without prescription” (ALSO), a pilot intervention to expand HIV testing through medical laboratories in France
Source: PLoS One. 2024 Oct 24;19(10):e0309754. doi: 10.1371/journal.pone.0309754 (PMC11500895; doi:10.1371/journal.pone.0309754)
Supplement: S4 Table — (DOCX) [file pone.0309754.s004.docx]

**S4 Table. Mean costs of HIV testing, by step and in total, according to test results, estimated by microcosting for an HIV test carried out in STI clinic**

| **STI clinic – NEGATIVE HIV test** | **(€)** |  | **STI clinic – POSITIVE HIV test** | **(€)** |
| --- | --- | --- | --- | --- |
| **HIV testing in STI clinic** | **36.70** |  | **HIV testing in STI clinic** | **94.01** |
| Admission | 1.69 |  | Admission | 1.69 |
| Pre-test counselling | 8.67 |  | Pre-test counselling | 8.67 |
| Blood sampling | 6.65 |  | Blood sampling | 6.65 |
| Combined ELISA/AgP24 analysis | 13.97 |  | Combined ELISA/AgP24 analysis | 13.97 |
| Western blot analysis ^1^ | 0.27 |  | Western blot analysis | 42.66 |
| Result delivery and post-test counselling | 5.45 |  | Result delivery and post-test counselling | 11.78 |
|  |  |  | Psychologist support ^2^ | 8.60 |
| **Mean cost for one negative HIV test in STI clinic** | **36.70** |  | **Confirmatory analysis on a second sample** | **22.30** |
| ^1^ The specificity of combined ELISA/ AgP24 detection being 99.8%, a Western blot was carried out for 0.2% of negative tests.  ^2^ Acceptance rate of psychologist support at the time of the positive result delivery: 50%.  ^3^ First HIV care consultation is considered as complex consultation, the cost of a specialist physician visit of €30 is increased by €30. |  |  | Admission | 1.69 |
|  |  |  | Blood sampling | 6.65 |
|  |  |  | Combined ELISA/AgP24 analysis | 13.97 |
|  |  |  | **Consultation to a specialised HIV unit**^3^ | **60.00** |
|  |  |  | **Mean cost for one positive HIV test in STI clinic** | **176.32** |

In STI clinics, counselling and result delivery were carried out by physicians (except in one centre where nurses delivered negative results) and sampling, by nurses. In contrast, for testing with a rapid test, nurses were more likely to be involved in the counselling and rapid testing and negative test delivery.
