# Supplementary material for: Facilitating the access to HIV testing at lower costs: “To the laboratory without prescription” (ALSO), a pilot intervention to expand HIV testing through medical laboratories in France
Source: PLoS One. 2024 Oct 24;19(10):e0309754. doi: 10.1371/journal.pone.0309754 (PMC11500895; doi:10.1371/journal.pone.0309754)
Supplement: S5 Table — (DOCX) [file pone.0309754.s005.docx]

**S5 Table. Mean costs of HIV testing, by step and in total, according to test results, estimated by microcosting for a rapid HIV test carried out in STI clinic**

| **STI clinic – NEGATIVE rapid HIV test** | **(€)** |  | **STI clinic – POSITIVE rapid HIV test** | **(€)** |
| --- | --- | --- | --- | --- |
| **Rapid HIV testing in STI clinic** | **18.94** |  | **Rapid HIV testing in STI clinic** | **28.48** |
| Admission | 1.69 |  | Admission | 1.69 |
| Pre-test counselling | 6.54 |  | Pre-test counselling | 6.54 |
| Rapid HIV testing | 8.98 |  | Rapid HIV testing | 8.98 |
| Result delivery and post-test counselling | 1.73 |  | Result delivery and post-test counselling | 11.27 |
| **Mean cost for one negative rapid HIV test in STI clinic** | **18.94** |  | **Confirmatory analysis** | **64.96** |
|  |  |  | Admission | 1.69 |
|  |  |  | Blood sampling | 6.65 |
| ^1^ First HIV care consultation is considered as complex consultation, the cost of a specialist physician visit of €30 is increased by €30. |  |  | Combined ELISA/AgP24 analysis + Western blot analysis | 56.63 |
|  |  |  | **Consultation to a specialised HIV unit**^1^ | **60.00** |
|  |  |  | **Mean cost for one positive rapid HIV test in STI clinic** | **153.44** |
