# Supplementary material for: Facilitating the access to HIV testing at lower costs: “To the laboratory without prescription” (ALSO), a pilot intervention to expand HIV testing through medical laboratories in France
Source: PLoS One. 2024 Oct 24;19(10):e0309754. doi: 10.1371/journal.pone.0309754 (PMC11500895; doi:10.1371/journal.pone.0309754)
Supplement: S6 Table — (DOCX) [file pone.0309754.s006.docx]

**S6 Table. Mean costs of HIV testing, by step and in total, according to test results, estimated by microcosting for a rapid HIV test carried out in community-based organisation (CBO)**

| **CBO – NEGATIVE rapid HIV test** | **(€)** |  | **CBO – POSITIVE rapid HIV test** | **(€)** |
| --- | --- | --- | --- | --- |
| **Rapid HIV testing in CBO** | **40.31** |  | **Rapid HIV testing in CBO** | **54.15** |
| Admission | 5.56 |  | Admission | 5.56 |
| Pre-test counselling | 19.84 |  | Pre-test counselling | 19.84 |
| Rapid HIV testing |  |  | Rapid HIV testing |  |
| Result delivery and post-test counselling | 3.73 |  | Result delivery and post-test counselling | 17.58 |
| Outreach actions fees | 11.18 |  | Outreach actions fees | 11.18 |
| **Mean cost for one negative rapid HIV test in CBO** | **40.31** |  | **Confirmatory analysis in STI clinic** | **76.74** |
|  |  |  | Admission | 1.69 |
|  |  |  | Blood sampling | 6.65 |
| ^1^ First HIV care consultation is considered as complex consultation, the cost of a specialist physician visit of €30 is increased by €30. |  |  | Combined ELISA/AgP24 analysis + Western blot analysis | 56.63 |
|  |  |  | Result delivery and post-test counselling | 11.78 |
|  |  |  | **Consultation to a specialised HIV unit**^1^ | **60.00** |
|  |  |  | **Mean cost for one positive rapid HIV test in CBO** | **190.90** |

On average, 80% of the workers who reached out to and received people who wanted to be tested and 20% of those who carried out counselling and testing were volunteers. From the collective perspective, the time volunteers devoted to community-based HIV testing has not been valued.
